# Functional Connectivity at Rest between the Human Medial Posterior Parietal Cortex and the Primary Motor Cortex Detected by Paired-Pulse Transcranial Magnetic Stimulation

**DOI:** 10.3390/brainsci11101357

**Published:** 2021-10-15

**Authors:** Rossella Breveglieri, Sara Borgomaneri, Matteo Filippini, Marina De Vitis, Alessia Tessari, Patrizia Fattori

**Affiliations:** 1 Department of Biomedical and Neuromotor Sciences, University of Bologna, 40126 Bologna, Italy; matteo.filippini7@unibo.it (M.F.); marina.devitis@unibo.it (M.D.V.); patrizia.fattori@unibo.it (P.F.); 2 Center for Studies and Research in Cognitive Neuroscience, University of Bologna, 47521 Cesena, Italy; sara.borgomaneri@unibo.it; 3 IRCCS Santa Lucia Foundation, 00179 Rome, Italy; 4 Department of Psychology “Renzo Canestrari”, University of Bologna, 40127 Bologna, Italy; alessia.tessari@unibo.it; 5 Alma Mater Research Institute for Human—Centered Artificial Intelligence (Alma Human AI), University of Bologna, 40126 Bologna, Italy

**Keywords:** medial posterior parietal cortex, functional connectivity, transcranial magnetic stimulation, paired pulse stimulation, parieto-M1 network

## Abstract

The medial posterior parietal cortex (PPC) is involved in the complex processes of visuomotor integration. Its connections to the dorsal premotor cortex, which in turn is connected to the primary motor cortex (M1), complete the fronto-parietal network that supports important cognitive functions in the planning and execution of goal-oriented movements. In this study, we wanted to investigate the time-course of the functional connectivity at rest between the medial PPC and the M1 using dual-site transcranial magnetic stimulation in healthy humans. We stimulated the left M1 using a suprathreshold test stimulus to elicit motor-evoked potentials in the hand, and a subthreshold conditioning stimulus was applied over the left medial PPC at different inter-stimulus intervals (ISIs). The conditioning stimulus affected the M1 excitability depending on the ISI, with inhibition at longer ISIs (12 and 15 ms). We suggest that these modulations may reflect the activation of different parieto-frontal pathways, with long latency inhibitions likely recruiting polisynaptic pathways, presumably through anterolateral PPC.

## 1. Introduction

Parieto-frontal networks are actively involved in monitoring arm movements such as reaching and grasping in monkeys [1,2,3,4,5,6,7] and in humans [8,9,10,11,12,13,14,15,16]. In the monkey brain, area V6A of the medial posterior parietal cortex contains reaching [17,18] and grasping cells [19,20,21,22]. Given the knowledge of monkey V6A connections with the occipital, parietal, mesial and frontal cortices [23,24,25], area V6A may integrate sensory and motor-related input to estimate the limb state during arm movement, and participates in the exchange of information with the frontal cortex, specifically with the dorsal premotor cortex (PMd), which is in turn connected to the primary motor cortex (M1), and this is necessary to perform accurate interactions with objects in the peripersonal space [2,26,27].

Area V6A also exists in humans (hV6A) [28] and is connected with the PMd via the superior longitudinal fasciculus [29,30]. Using resting-state fMRI, the same patterns of connections of hV6A were found in humans [8].

Given the fundamental importance of such parieto-frontal connections in orchestrating our movements, it appears to be crucial to investigate the time course of interactions between hV6A and M1 in healthy humans. Moreover, this knowledge represents a fundamental step for a deeper understanding of possible dysfunctions of such connections in pathological conditions. Interestingly, it has been reported that latency of the mutual information exchange is altered in neurological conditions such as Parkinson’s disease and mirror dystonia [31,32].

As neuroanatomical studies in monkeys based on tracer injections cannot give information about the timing of connections, and fMRI studies rely on a correlational approach characterized by low temporal resolution, non-invasive brain stimulation techniques such as transcranial magnetic stimulation (TMS) appear better suited for disclosing the time-course of hV6A–M1 causal interactions. Specifically, the dual-site TMS paired-pulse protocol (ppTMS) was successfully used for non-invasively mapping causal connectivity with high temporal resolution [11,12,33,34,35,36,37,38,39,40]. In the ppTMS protocol, a conditioning stimulus (CS) is administered over a target (e.g., parietal) area to activate direct or indirect pathways from the target site to M1. The CS is followed by a test stimulus (TS) administered over M1 to induce motor-evoked potentials (MEPs) in contralateral muscles. Both facilitation and inhibition may occur at the TS site (i.e., M1) depending on CS intensity and the interstimulus intervals (ISIs) between CS and TS [13,38].

The timing of the functional connections within the human parieto-frontal network was studied with ppTMS. Specifically, it has been found that when the CS administered in a region (superior parieto-occipital cortex, SPOC) that partially overlaps with the hV6A [10] precedes TS by 4 ms, a MEP facilitation was observed during reach-to-grasp planning in the first dorsal interosseous (FDI) [11], whereas when CS precedes TS by 6 ms, the same facilitation on a grasp-related muscle (abductor digiti minimi, ADM) was observed during whole-hand grasp planning [12].

The functional connectivity within the parieto-M1 network has been investigated not only during the planning of reaching and grasping movements, but also at rest. The seminal work of Koch demonstrated that a facilitation of MEPs can be obtained by a CS administered on the lateral posterior parietal cortex (PPC) preceding TS by 4, 6 and by 15 ms [13], depending on the hemisphere. However, when targeting the lateral PPC, different results have been obtained depending on the hemisphere and the site of the CS stimulation [41,42]. When considering other medial parietal sites, no modulations of MEPs evoked by the TS were observed; neither when the CS was delivered in area 5 [43] nor when it was administered in more posterior sites, such as SPOC [11]. Thus, different and inconsistent results have been obtained when investigating lateral PPC–M1 functional interactions at rest. Moreover, functional interactions between the medial PPC and the M1 are still largely unexplored. Specifically, ISIs longer than 10 ms have never been tested for medial PPC–M1 interactions; thus, the novelty of the present study is to provide new evidence about functional interactions involving medial PPC–M1 with longer latency, which can offer novel insights into clinical conditions associated with altered connectivity patterns.

Thus, we systematically studied the functional interactions between the medial PPC area hV6A and the M1 at rest using different ISIs ranging from 4 ms to 15 ms. Our findings show a time-dependent modulation of the hV6A–M1 connectivity during a resting state, with long latency (12–15 ms) inhibition which likely reflects the recruitment of polysynaptic circuits.

## 2. Materials and Methods

### 2.1. Participants

Fourteen healthy volunteers (seven men, aged 19–34 years old) participated in this study. The number of participants is comparable with the sample size determined by a power analysis ((1–β) of 0.95; 2-tailed α = 0.05; effect size f = 0.25; number of measurements = 14; correlation = 0.5, analysis performed with G*Power software [44]).

All the participants were right-handed according to a standard handedness inventory [45], and had normal or corrected-to-normal visual acuity in both eyes. None of the participants had neurological, psychiatric, or other medical problems or any contraindication to TMS. Participants provided written informed consent, the procedures were approved by the Bioethical Committee at the University of Bologna (Prot. 57635, 11 March 2021), and were in accordance with the ethical standards of the Declaration of Helsinki (2013). No discomfort or adverse effects during TMS were reported or noticed.

### 2.2. Localization of Brain Sites

**Figure 1:**
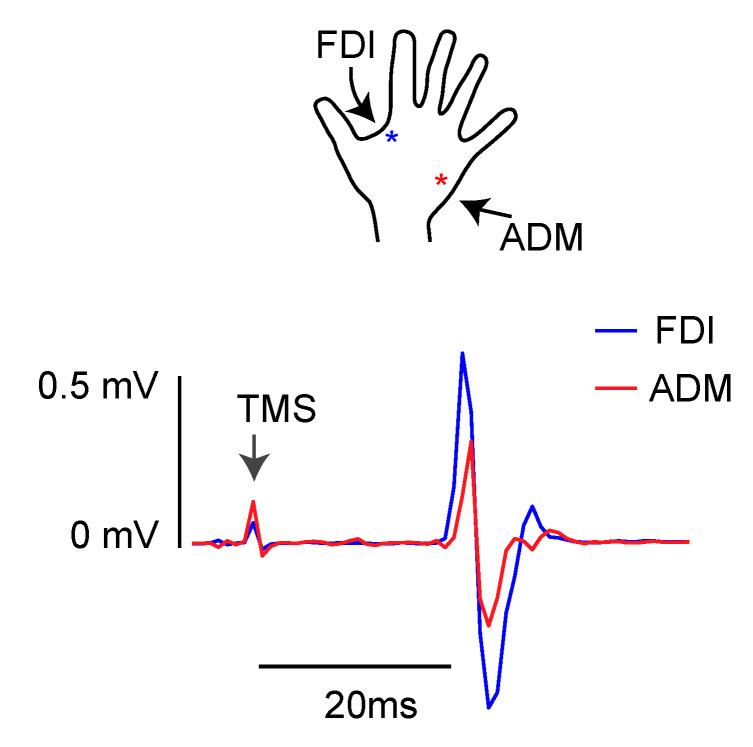
Typical EMG recording for FDI (blue trace) and ADM (red trace) muscle activity during rest. During the stimulation (TMS), artifacts on EMG trace are also shown.

Before each experimental session, the positions of the coils were identified on each participant’s scalp. The optimal scalp position for coil placement over the left M1 was defined on the participant’s head wearing a bathing cap, as the point where stimulation evoked the largest MEPs from the contralateral first dorsal interosseous (FDI) muscle of the right hand (Figure 1). To identify area hV6A in the left hemisphere, we used frameless stereotaxic neuronavigation before each experimental session using the SofTaxic Navigator system (E.M.S. srl, Bologna, Italy) [46,47,48]. In the first stage, skull landmarks (nasion, inion, and 2 preauricular points) and 65 points providing a uniform representation of the scalp, were digitized by means of a Polaris Vicra Optical Tracking System (Northern Digital, Inc., Waterloo, ON, Canada). Coordinates in Talairach space were automatically estimated by the SofTaxic Navigator from an MRI-constructed stereotaxic template. This procedure has been proven to ensure a good localization accuracy, showing an error of roughly 5 mm in comparison to methods based on individual MRIs [48].

The Talairach coordinates of hV6A we used were x = −10, y = −78, and z = 40 [49]. These coordinates are the same as those used in two previous TMS studies on hV6A [50,51], and are similar to those used for studying area SPOC [12,52], a region also investigated in imaging studies [8,10,53] that likely includes hV6A [54]. Then, the neuronavigation system was used to estimate the projections of scalp sites on the brain surface. Mean coordinates ± standard deviation corresponded to the hV6A (x = −12.49 ± 0.62 y = −79.85 ± 3.94 z = 38.92 ± 3.97).

### 2.3. Transcranial Magnetic Stimulation

A dual-site, paired-pulse transcranial magnetic stimulation paradigm with two coils was used to test connectivity between the left PPC (hV6A) and the left M1. TMS pulses were administered via two T-shaped 50 mm butterfly coils, each of which was connected to a DuoMAG MP-Dual TMS System monophasic transcranial stimulator (DEYMED, Hronov, Czech Republic).

To set TMS intensity, the resting motor threshold (rMT) was estimated for all participants in a preliminary phase of the experiment using standard procedures [55]. MEPs induced by stimulation of the left motor cortex were recorded from the right first dorsal interosseous (FDI) and from the abductor digiti minimi (ADM) by means of a 2-channel DuoMAG MEP amplifier. Electromyography (EMG) signals were FIR-filtered and digitized at a sampling rate of 5 kHz. Pairs of disposable pre-gelled Ag–AgCl surface electrodes were placed in a belly-tendon montage with a ground electrode on the wrist. The optimal scalp position for inducing MEPs from the right FDI was first localized, and the rMT was determined from that position. The rMT was defined as the minimal intensity of stimulator output that produced MEPs with an amplitude of at least 50 μV in the FDI with a 50% probability [56]. The mean rMT across participants was 43.79%, in line with other studies [57].

We administered TMS as the TS over M1 and the TS coupled with a preceding CS over hV6A with 6 ISIs (4, 6, 8, 10, 12, 15 ms). We divided the stimulations into 3 blocks. In the first block, we tested the TS alone (s-pulse) or coupled with the CS (*p*-pulse) with 4 and 10 ms as ISIs; in the second block, the s-pulse and *p*-pulse were tested with ISIs of 6 and 8 ms; in the third block, s-pulse and *p*-pulse were tested with 10 and 15 ms as ISIs. The order of the blocks, and the order of the pulses within each block, was randomized across participants. Each block consisted of 47 trials (7 TS trials, 20 CS-TS at each ISI) with a fixed inter-trial interval of 6 s. A 3 min break was allowed between blocks. Participants sat on a comfortable chair in a darkened room and their head was kept stable using a head/chin rest. They were asked to keep both hands relaxed while testing, with the aim of obtaining a stable EMG signal. The intensity of TS was adjusted to elicit a motor-evoked potential (MEP) of 1 mV peak to peak in the relaxed right FDI [13], and this corresponded to 120.32% of the rMT across participants. The intensity of the CS stimulus was set at 90% rMT [11,12,13]. Both coils were held tangential to the skull, with the M1 coil at 45° and hV6A coil at 90° from the mid-sagittal line to induce a posterior–anterior directed current in the underlying cortical tissue [11,12].

### 2.4. Electromyographic Recordings

During each stimulation session, EMG was used to monitor muscle activity from FDI and from ADM. Surface electromyograms were recorded with 9 mm diameter, Ag–AgCl surface-cup electrodes. EMG signals were recorded by means of a Digitimer D440-4 system (Digitimer, Welwyn Garden City, Hertfordshire, UK), amplified to 1000×, band-pass-filtered between 30 Hz and 1 kHz with a sample rate of 5 KHz, recorded using a Micro1401 data acquisition interface controlled by Signal software v7 (Cambridge Electronic Design Ltd., Cambridge, UK), and stored on a computer for off-line analysis.

### 2.5. Data Analysis

The mean peak-to-peak MEP amplitude was computed for the s-pulse and *p*-pulse condition in each block. We checked for any trace showing EMG activity 100 ms prior to the TMS pulses and, in each condition, for any MEPs with amplitudes deviating from the mean by more than 2.5 standard deviations. No MEPs were discarded based on this analysis.

We averaged the MEP obtained in the s-pulse conditions of the 3 blocks because their MEPs were not statistically different between blocks (2-way ANOVA Muscle x Block, all *p* > 0.46). Thus, a two-way analysis of variance (ANOVA, with Greenhouse–Geisser correction for nonsphericity, Mauchly’s test *p* < 0.05) with factors Muscle (2 levels, FDI and ADM), and TMS (7 levels, s-pulse, *p*-pulse4, *p*-pulse6, *p*-pulse8, *p*-pulse12, *p*-pulse15) was performed with the peak-to-peak MEP amplitude as the dependent variable. A post-hoc analysis was performed with the Newman–Keuls test in order to compare MEPs of the different conditions, and to correct for multiple comparisons. The significance level was set at 0.05.

## 3. Results

We recorded MEPs from FDI and ADM muscles at rest in different blocks, with the aim of studying the time course of the resting functional connectivity between hV6A and M1.

**Figure 2:**
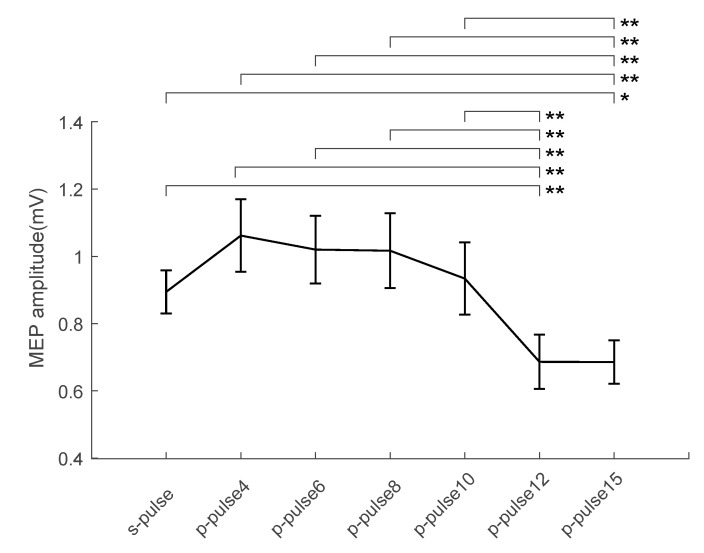
Physiological interactions between PPC and ipsilateral M1 at rest. Group-averaged (*n* = 14), muscle-averaged MEP amplitude (mV) for each ISI (*p*-pulse) including TS alone (s-pulse) during rest. Lines above the bars represent significant differences (Newman–Keuls post-hoc comparisons, * = *p* < 0.05, ** = *p* < 0.01) between conditions. Error bars represent the standard error.

**Figure 3:**
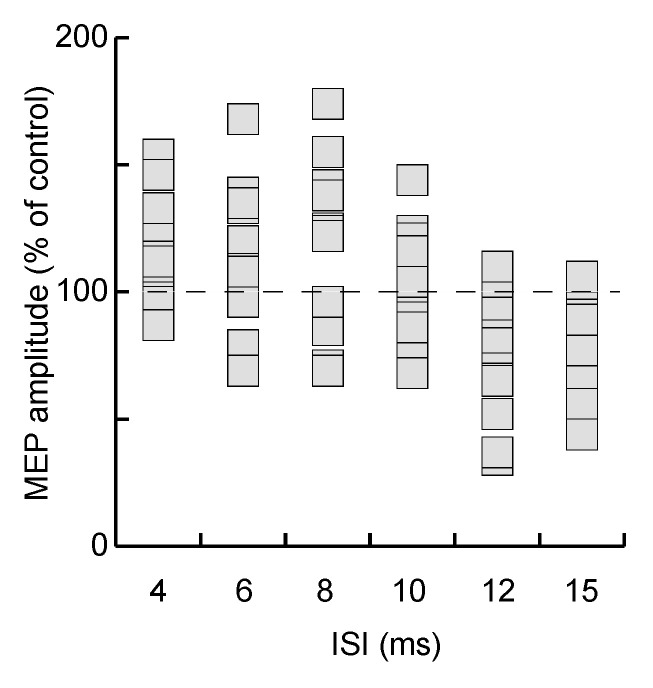
Individual participants’ MEP amplitudes at the different ISIs. MEP amplitude is expressed as the percentage of the control condition (s-pulse). The dashed horizontal line represents 100% (i.e., when the *p*-pulse condition equals the s-pulse condition). Each rectangle represents the mean MEP amplitude across trials in each participant.

The functional connectivity between hV6A and M1 followed a specific time course, as demonstrated by the modulation of MEP amplitude by the CS. Specifically, MEP amplitude was influenced by TMS (F_(6,78)_ = 8.11, *p* < 0.001, η_p_^2^ = 0.38; Figure 2; mean values in Table 1; individual participants’ data in Figure 3). This effect was driven by the reduction in MEP amplitude when ISI was longer than 10 ms. This inhibition was significant at ISIs of 12 ms (*p* < 0.01) and 15 ms (*p* = 0.02, Figure 2 and Figure 3).

MEP amplitude was also affected by the muscle (F_(1,13)_ = 16.28, *p* < 0.001, η_p_^2^ = 0.55), with MEP recorded from FDI larger than those recorded from ADM (*p* < 0.001). On the contrary, the interaction of the factors muscle and TMS was not significant (F_(6,78)_ = 2.93, *p* = 0.06, η_p_^2^ = 0.18), suggesting that the activation of the two muscles followed the same trend.

## 4. Discussion

In this work, we demonstrated that hV6A impacts ipsilateral M1 excitability at rest. This modulation was time-dependent, with significant inhibition for ISIs longer than 10 ms. These modulations imply that the two regions are functionally connected. Monkey and human studies have established that the connections between V6A and M1 are mainly indirect [8,23,24]. The shorter route from V6A to the frontal cortex is the connection with the PMd, which in turn is connected to the M1 [8,23]. In humans, specifically, the connections between the superior parietal lobule and PMd lie within the first branch of the superior longitudinal fasciculus [29,30,58]. In contrast, anatomical evidence of a direct connection between the medial PPC and M1 is still lacking. We suggest that the inhibition we found at long ISIs could be the result of the activation of polysynaptic routes between hV6A and the frontal cortex. These longer pathways could involve either the connections between hV6A and PMd-M1, likely involving one or more interneurons within PMd and/or M1, or the direct connection of hV6A with the antero-lateral intraparietal regions (areas VIP, LIP and AIP) [8,23], which in turn are connected directly to the M1 [59,60,61] or the spinal cord [42,62], or even more indirectly via the ventral premotor cortex [61]. This latter suggestion is supported by the inhibition found after conditioning the M1 with a CS in the antero-lateral intraparietal regions at ISIs between 2 and 6 ms [41].

### Comparisons with Other Studies about Parietal Resting Connectivity

Resting state connectivity has extensively been tested with ppTMS in the human parieto-M1 network. When CS was administered over the lateral parietal cortex, different results were obtained according to the hemisphere tested [13,41], with inhibitory effects when CS preceded TS by 2–6 ms in different loci around the right intraparietal sulcus (IPS). Conversely, in the left hemisphere, inhibition followed the same time-course only when the CS was administered in the anterior IPS and an excitatory effect was observed when the CS was in the central and posterior IPS [41]. The inhibition for a 4 ms ISI in the left lateral anterior IPS was also confirmed by Vesia [11]. When CS was administered over the right posterior IPS, excitatory effects were observed at ISIs of 4 and 15 ms, whereas in the left hemisphere, the same facilitation was found at ISIs of 4 and 6 ms [13]. Even if a strict comparison of the abovementioned results is difficult given the differences in the stimulation sites, the emerging frame about the left hemisphere is that after stimulation of anterolateral PPC, inhibitory effects over M1 are exerted, whereas excitatory effects are evident after the stimulation of posterolateral PPC [13,41]. Cattaneo and collaborators, on the contrary, found only inhibitory effects when CS was delivered on the lateral part of the superior parietal lobule [42]; however, this study was performed in anaesthetized brain tumor patients, so they may be not fully comparable with the results obtained in awake and healthy participants.

Differently from the abovementioned studies, our CS stimulation site was in the medial PPC, specifically in hV6A. Given the small extension of hV6A and the spread of the stimulation with a 50 mm coil, we cannot rule out the possibility that a spread of stimulation over neighboring areas occurred, namely, in the lateral portion of posterior Brodmann’s area 7. However, the coil was centered on hV6A, and we delivered single pulses. The possible spread of the single pulse stimulation over neighboring areas would have only been in low intensity levels [63].

The anterior part of the medial PPC, specifically, the medial part of Brodmann’s area 5, located rostrally to hV6A [54,64], did not have any effect on M1-evoked MEPs [43]. By administering CS more posteriorly, in the anterior part of Brodmann’s area 7, Karabanov [65] found the facilitation of MEPs at 2 ms ISIs. Even more caudally, Vesia did not find any effect on M1 excitability [11] when CS was delivered on SPOC, a region that partially overlaps with our current stimulation site. This result corroborates what we found here, at least for ISIs from 4 to 10 ms, which were tested in the current study and that of Vesia [11], where the observed effects did not reach the statistical threshold for significance.

The current study extends the results of Vesia by adding two longer ISIs in the experiments, and by demonstrating, for the first time, inhibitory effects of hV6A over M1 observed at longer ISIs.

## 5. Conclusions

Our experiments demonstrate the first evidence of a slow 12–15 ms inhibitory intra-hemispheric cortico-cortical circuit between hV6A and M1. We suggest that the connectivity at rest of hV6A with the M1 may involve the antero-lateral intraparietal cortex, or interneurons within the premotor cortex and/or M1. How these connections can be recruited during reaching and grasping planning will be the object of future studies.

## Figures and Tables

**Table 1:** Mean values and standard error of the MEP amplitude in the different TMS conditions.

TMS	Mean	Standard Error
s-pulse	0.89	0.06
*p*-pulse4	1.06	0.10
*p*-pulse6	1.02	0.10
*p*-pulse8	1.02	0.11
*p*-pulse10	0.93	0.11
*p*-pulse12	0.69	0.08
*p*-pulse15	0.69	0.06

## Data Availability

The data presented in this study are available on reasonable request from the corresponding author.
